# A high-performance, all-solid-state Na^+^ selective sensor printed with eco-friendly conductive ink†

**DOI:** 10.1039/d3ra01410j

**Published:** 2023-06-06

**Authors:** Dengke Wang, Wanggang Zhang, Jian Wang, Xiaohong Li, Yiming Liu

**Affiliations:** a College of Chemical Engineering and Technology, Taiyuan University of Technology Taiyuan Shanxi 030024 China; b College of Materials Science and Engineering, Taiyuan University of Technology Taiyuan Shanxi 030024 China; c Shanxi Academy of Analytical Sciences Taiyuan 030006 Shanxi China; d State Key Laboratory of Clean and Efficient Coal Utilization, Taiyuan University of Technology Taiyuan Shanxi 030024 China

## Abstract

In recent years, the integration of flexible printed electronics and electrochemical sensors has emerged as a new approach for developing wearable biochemical detecting devices. Among the materials utilized in flexible printed electronics, carbon-based conductive inks are considered to be crucial. In this study, we propose a cost-effective, highly conductive, and environmentally friendly ink formulation utilizing graphite and carbon black (CB) as conductive fillers, resulting in a very low sheet resistance of 15.99 Ω sq^−1^ (conductivity of 2.5 × 10^3^ S m^−1^) and a printed film thickness of 25 μm. The unique “sandwich” structure of the working electrode (WE) printed with this ink enhances its electrical conductivity, leading to high sensitivity, selectivity, and stability, with almost no water film generated between the WE and the ion-selective membrane (ISM), strong ion selectivity, long-term stability, and anti-interference. The lower detection limit of the sensor for Na^+^ is 0.16 mM with a slope of 75.72 mV per decade. To validate the sensor's usability, we analyzed three sweat samples collected during physical activity, with Na^+^ concentrations within the typical range for human sweat (51 ± 4 mM, 39 ± 5 mM, and 46 ± 2 mM).

## Abstract 2

A highly conductive ink printing sensor for sweat detection.

## Graphical abstract

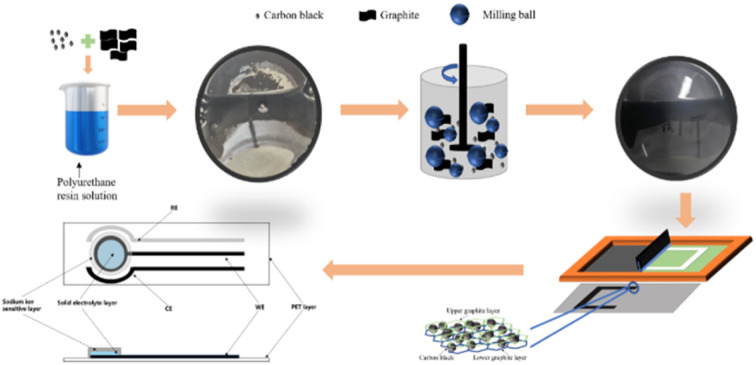

## Introduction

Ion-selective sensors (ISS) play a crucial role in advancing analytical chemistry and are the driving force behind the creation of electrochemical sensors, which can detect various sample solutions non-destructively without requiring external energy to neutralize ion activity. Due to their low production cost, ease of operation, sensitive response, and rapid response,^1–4^ ISS is widely used to detect ions in fields such as biomedicine, the environment, and industry.^5–10^ All-solid-state ISS (ASS-ISS), which can be created on a wide scale *via* printing, is a developing flexible electronic technology with great performance that is downsized and portable when compared to traditional ISS. This method provides the possibility of inventing a new type of potential sensor with minimal maintenance cost and good stability.

Currently, the sensing mechanisms of ASS-ISS mainly include double-layer capacitance and pseudo-capacitance types. The sensing mechanism of a double-layer capacitive sensor is to form a double-layer capacitor similar to an asymmetric capacitor between the solid-state conductive layer and the ion-selective film (ISM).^11,12^ The use of a large capacitor can increase the contact area between the solid-state conductive layer and the ISM, stabilize the interface potential, and improve electron transfer efficiency. A double-layer capacitive sensor can be described as an asymmetric electronic capacitor. One side is the charge carried by the target cation or anion passing through the ISM, and the other side is the electronic charge formed by the electrons or holes in the conductive layer. This type of conductive layer material mainly consists of carbon nanomaterials and metal nanoparticles. The magnitude of the potential depends on the total amount of charge in the double layer.^13,14^ ASS-ISS prepared from a conductive material with a large redox capacitor is called a pseudo-capacitive type solid-state ion selective sensor. This type of material has both electronic conductivity and excellent ion conductivity due to ion doping. Its response mechanism is to transfer charge from ions to electrons through an oxidation–reduction reaction, then through the conductive substrate, and finally obtain the ion concentration by displaying the voltage.^15^ Nevertheless, the water layer and the charge-transfer resistance between the solid-state conductive layer and the ISM prevents ASS-ISS from being used on a large scale.

Conductive ink is one of the most crucial materials in the field of printed electronics.^16^ Conductive coatings made from traditional carbon materials like graphite and CB, as well as newer carbon materials like graphene and carbon nanotubes, offer excellent electrical conductivity, corrosion resistance, and environmental friendliness, among other benefits, for a variety of printed electronics applications such as batteries and supercapacitors, electrochemical sensors, printed circuit board resistors, printed heaters, and solar energy harvesting.^17–22^ Screen printing is the primary ink processing method for these applications, and it has attracted widespread attention in the field of flexible electronics due to its low cost, ease of mass manufacturing, and powerful design.^23,24^ Several types of publications have reported on the application of screen-printing technology to electrochemical devices. V. Mazzaracchio *et al.*^25^ developed a wireless flexible device for monitoring sweat pH by electroplating an iridium oxide thin film over a graphite WE, which provided a pH-sensitive layer and measured the pH in the solution. The slope of the response was −0.079 ± 0.002 V per decade. The same research group also introduced a novel type of screen-printed electrode,^1^ in which the WE was modified by drop-casting a combination of nanomaterial CB and a sodium ISM. The electrode has a detection range of 10^−4^ M to 1 M for Na^+^ and a response slope of 58 ± 3 mV per decade. A. Hauke *et al.*^26^ created a screen-printed, low-cost chloride ion sensor for remote monitoring of cystic fibrosis (CF) patients with high sensitivity and a detection limit of 2.7 × 10^−5^ M, covering the entire concentration range for CF diagnostics and available as a portable device at a greatly reduced cost. Furthermore, several literature publications^27–29^ have demonstrated that screen printing technology can manufacture electrochemical sensors with cheap cost, mobility, large-scale manufacturing, and outstanding performance, which can be used in biosensing, environmental detection, and analysis.

Carbon materials play a crucial role in determining the microstructure and physical properties of WE. The pore structure and high specific surface area of carbon materials have a direct impact on the transmission efficiency of the electrode, thereby enhancing the analytical performance of screen-printed electrodes (SPE).^30–32^ Additionally, the physical and chemical properties of carbon materials facilitate the stable passage of electrodes. Building upon this understanding, we have developed a novel method for manufacturing SPE capable of detecting Na^+^ concentrations. Our approach employs conductive ink comprising graphite and CB, and screen-printing technology to fabricate carbon electrodes. Based on the foregoing, we disclosed a new technique for manufacturing a screen-printed electrode for detecting Na^+^ concentration, conductive ink made from graphite and CB, and a carbon electrode made using screen printing technology (Fig. S1†). It has great conductivity and high capacity and may be employed as a suitable conductive substrate, thus improving ASS-ISS's sensitivity, potential stability, and dependability.

The potential difference, according to the Nernst equation, is linear with the logarithm of the ion concentration to be measured. Human perspiration has a typical Na^+^ content of around 40 mM. We reported an All-solid-state sodium ion selective sensor (ASS-Na-ISS) in a NaCl concentration gradient solution. The linear fitting slope is 75.72 mV per decade when evaluating the potential difference between the ASS-Na-ISS and the reference electrode (RE). As far as we know, this is the first time a superior reaction has been found in earlier literature studies. Additionally, the electrode offers advantages such as long-term stability, excellent printing performance, low cost, and the ability for large-scale production. The performance of the electrode was evaluated in both Na^+^ solution and sweat, demonstrating its high potential for use in biosensing and detection. These findings broaden the scope of future applications for electrochemical flexible devices.

## Experimental section

### Materials

CB was purchased from Cabot Corporation. Graphite was obtained from Qingdao Jintao Graphite Co., Ltd. Defoamer (BYK-022) was purchased from BYK Chemistry. Dispersant (T-859) and waterborne polyurethane resin were purchased from Shenzhen Jitian Chemical Co., Ltd. PEDOT/PSS was obtained from 3AMaterial. Sodium ionophore III (ETH2120) was purchased from Shanghai Yuanye Biotechnology Co., Ltd (MedMol). Potassium tetrakis-(4-chlorophenyl)-borate (KTClPB) and bis-(2-ethylhexyl)-sebacate (DOS) was purchased from Aladdin. Polyvinyl chloride (PVC), cyclohexanone (CHA), and TritonX-100 were got from Shanghai Macklin Biochemical Co., Ltd. Ag/AgCl paste was purchased from Shanghai Julong Electronic Technology Co., Ltd. All chemicals and solvents were used as received without further treatment.

### Preparation of conductive ink

Table S1† shows the composition ratio for the production of 300 g of ink with a carbon content of 30% (graphite to CB ratio of 2 : 1 (ref. 33)). Mix the deionized water, water-based polyurethane resin, defoamer, and dispersant in a beaker, and then add graphite and CB to the mixture. To disperse the mixture by using a disperser for 4 h at 3000 rpm. Next, the dispersed ink is ball-milled for 6 h. Obtain samples every hour to investigate the effect of ball milling duration on electrical conductivity.

To explore the influence of carbon content on electrical conductivity, the carbon content was set as 20%, 25%, 28%, 30%, 32%, and 35% (w/w) in six groups (named G/C-20, G/C-25, G/C-28, G/C-30, G/C-32, G/C-35), the quality of other components remained unchanged.

### Printing of electrode

The printing plate pattern is designed, as shown in Fig. S2.† Installing the customized screen-printing plate on the screen-printing machine and using the scraper to squeegee the printing plate. The ink will be printed on the PET film through the screen to create the WE. The three electrodes, shown from left to right, are the RE, WE, and counter electrode (CE). The CE and WE were printed using ink, while the RE was printed using Ag/AgCl paste.

### Preparation of solid electrolyte layer

Take 1 mL PEDOT/PSS and mix it with 1‰ (V/V) TritonX-100. Leave the mixture at 4 °C for 4 h to ensure it is well mixed. Apply the mixture to the centre area of the WE of the printed electrode using a pipette gun. Allow the mixture to freely diffuse, and once it has covered the entire surface of the WE, place it in an oven and dry it at 40 °C to create a solid electrolyte layer.

### Preparation of Na^+^-ISM

Sodium ionophore III (1% w/w), ion exchanger (KTClPB, 0.1% w/w), high polymer (PVC, 33% w/w), and plasticizer (DOS, 65.9% w/w) combined with CHA (33 mg PVC per 350 μL CHA). Sonicate it for 10 minutes to ensure that it is evenly mixed, then let it aside for 1 hour before applying it onto the solid electrolyte layer with a pipettor. When the membrane liquid has completely covered the whole solid dielectric layer, dry the electrode in an oven at 40 °C to make a Na^+^-ISM. To prevent interference, apply insulating material to all exposed areas and save the electrodes.

### Instrumentation

Suzhou Jingge Electronics Co., Ltd's four-probe tester is used to test the conductivity (sheet resistance) of the ink. All electrochemical performance tests are carried out on the Autolab PGSTAT 302N produced by Metrohm. Field emission scanning electron microscopy (FE-SEM, TESCAN MIRA3 LMH) and transmission electron microscopy (TEM, JEOL2100) were used to observe the microstructure. Raman spectroscopy (Raman, Renishaw inVia) was used to characterize the internal structure.

## Results and discussion

Graphite is a layered material, and ball milling can thin the graphite layers to form few-layer graphite (a graphene-like structure). This can increase the conductivity of graphite and make it more similar to graphene,^34^ also can reducing the production cost. CB is a nano-scale conductive ball with a larger specific surface area than graphite. While graphite has high lateral conductivity on graphite sheets, its longitudinal conductivity between graphite sheets is substantially lower. In contrast, minute CB particles are more likely to scatter across graphite gaps and act as a bridge,^35^ generating a “sandwich” structure. So these two materials are more conductive when combined because the gaps between the conductive particles in this “sandwich” structure are smaller than either one alone. As a result, the conductive ink formed by combining these two materials has strong conductivity, which can effectively increase the transmission efficiency of the WE. This can lead to improved sensitivity and reaction time performance of the sensor. Additionally, carbon nanoparticles have a high specific surface area and are hydrophobic, which has been shown to improve potential drift, anhydrous layer interference, and the potential stability of electrochemical sensors.^36^

### Characterization and conductivity of the WE

**Fig. 1:**
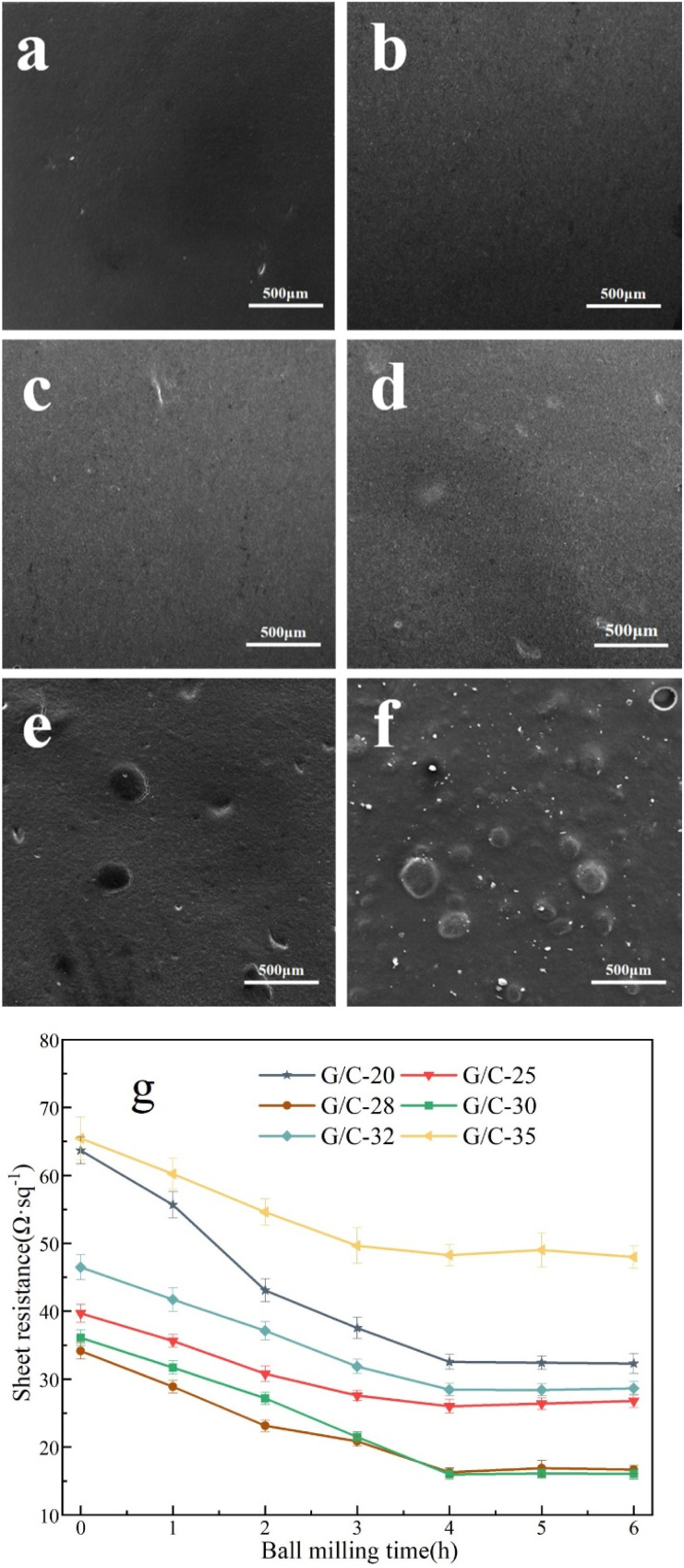
SEM images of (a) G/C-20, (b) G/C-25, (c) G/C-28, (d) G/C-30, (e) G/C-32 and (f) G/C-35 films at 100× magnification, (g) variation of sheet resistance of printed electrode with ball milling time.

In composite conductive inks, graphite and CB are used as conductive fillers, and their content has the greatest direct impact on the ink's conductivity. Fig. 1a–f shows the printed electrode surface images of six different carbon content inks observed by FESEM. When the carbon concentration is below 30%, the surface of the ink film is generally flat, and there is no agglomeration or uplift. However, when the carbon content is 32% and 35%, the surface of the ink film displays agglomeration and particles. This decrease in dispersibility suggests that the carbon content in the ink is oversaturated, resulting in some carbon fillers being unable to disperse well in the ink and agglomerating after printing. This increases thixotropy and leads to a decrease in surface flatness, which in turn reduces conductivity. Fig. 1g depicts the sheet resistance of six sets of printed electrodes with varying carbon content inks as a function of ball milling time, where 0 h represents the start of ball milling. The results indicate that when the ink ball milling duration grows during the early stage of ball milling, the sheet resistance decreases significantly. This is because the large particles in the ink evaporate, and the particle size of the conductive particles decreases (Fig. S3†), leading to a reduction in the contact resistance between the graphite and CB particles, and consequently, the sheet resistance drops.^37^ As the ball milling duration exceeds 4 h, the sheet resistance of the ink essentially remains unchanged. This may be due to that the ball milling process has reached its saturation point and the reduction in the contact resistance. Thus, the sheet resistance remains constant. The lowest sheet resistance was observed on the G/C-30 printed electrode after ball milling for 4 h, which is 15.99 Ω sq^−1^ (electrode thickness is 25 μm). According to the relationship between sheet resistance and conductivity:
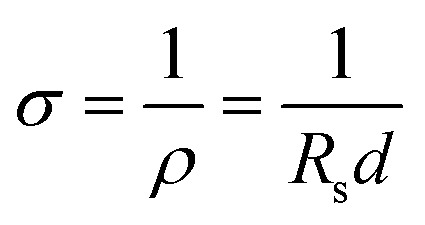


The conductivity is calculated as 2.5 × 10^3^ S m^−1^. *σ* is conductivity, *ρ* is resistivity, *R*_s_ is sheet resistance and *d* is the thickness of the electrode.

To summarize, it can be concluded that G/C-32 and G/C-35 inks are not ideal for printing due to the formation of agglomerates and uneven surfaces resulting from the oversaturation of carbon content, which can adversely affect performance. Therefore, further study on these inks will be avoided. On the other hand, G/C-30 ink showed the highest conductivity among the experimental groups and was chosen for the construction of ISS.

**Fig. 2:**
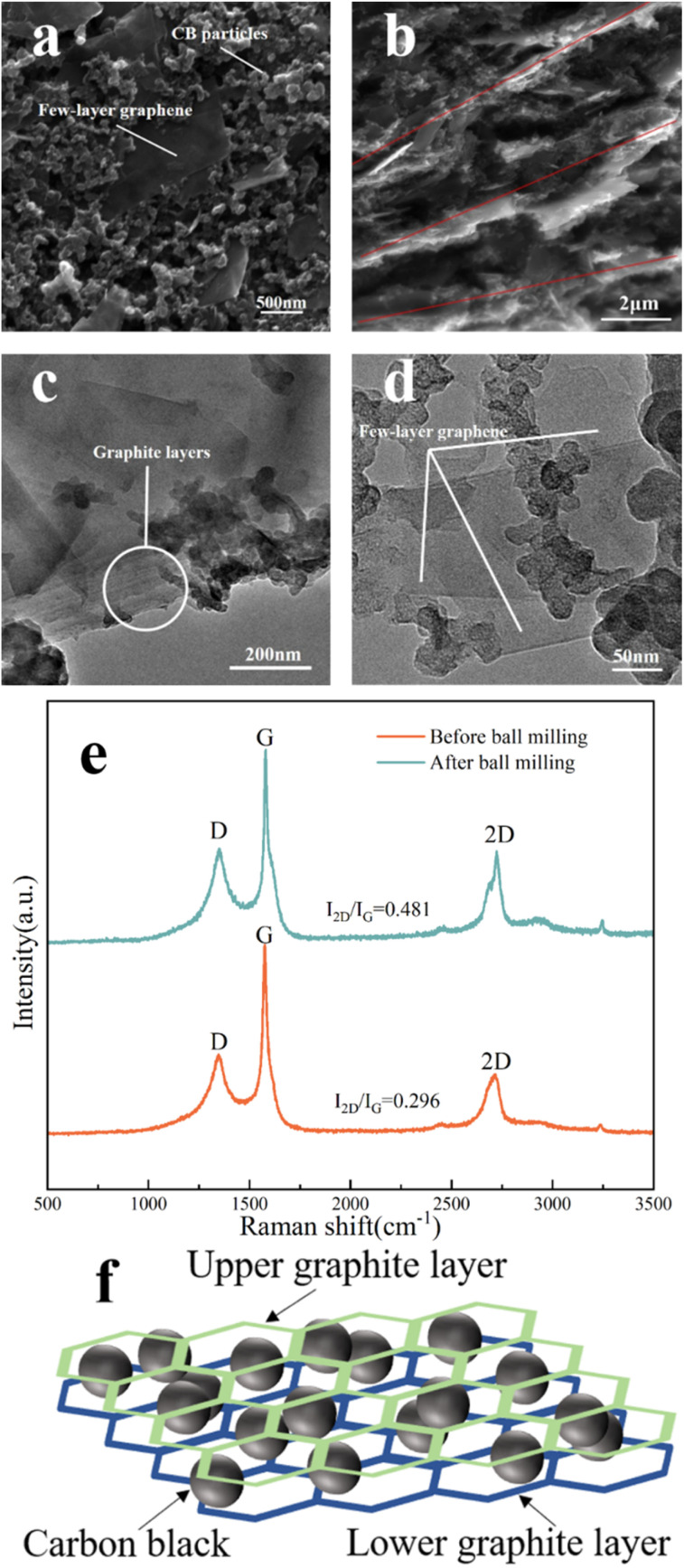
High magnification SEM image of G/C-30 printed electrode (a) 50k×, (b) 10k×; TEM image of G/C-30 printed electrode (c) before ball milling, (d) after ball milling; (e) Raman spectrum of G/C-30 ink before and after ball milling; (f) “sandwich” structure.

Fig. 2a presents Surface SEM images of the surface of the G/C-30 printed electrode, revealing that the CB in the ink is uniformly distributed between the graphite sheets, and these two carbon components are intimately mixed. The graphite and CB form a “sandwich” structure, as shown in Fig. 2b. Additionally, TEM characterizations of the diluted ink were performed before and after ball milling. In Fig. 2c, the graphite flakes in the diluted ink are multi-layered before ball milling. However, in the TEM image of the ink after ball milling (Fig. 2d), a graphene-like structure is visible, indicating the presence of transparent few-layer graphene.^38,39^ This conclusion also is demonstrated *via* Raman spectrum analysis. The Raman spectra of the conductive ink before and after ball milling is shown in Fig. 2e. The 2D peak of the ink grew dramatically after ball milling, and the *I*_2D_/*I*_G_ increased by 0.185. It has been demonstrated that the ball milling technique mechanically exfoliates graphite during the creation of conductive inks, resulting in the few-layer graphene.^40^ The characterization results indicate that ball milling can effectively disperse the CB particles in the ink, resulting in an even distribution among the graphite flakes and the formation of a “sandwich” structure. This leads to a reduction in contact resistance between the graphite flakes and an increase in conductivity between them. Additionally, ball milling can increase the number of graphite layers, resulting in the generation of few-layer graphene, which significantly improves the ink's conductivity.

### Cyclic voltammetry and electrochemical impedance spectroscopy

**Fig. 3:**
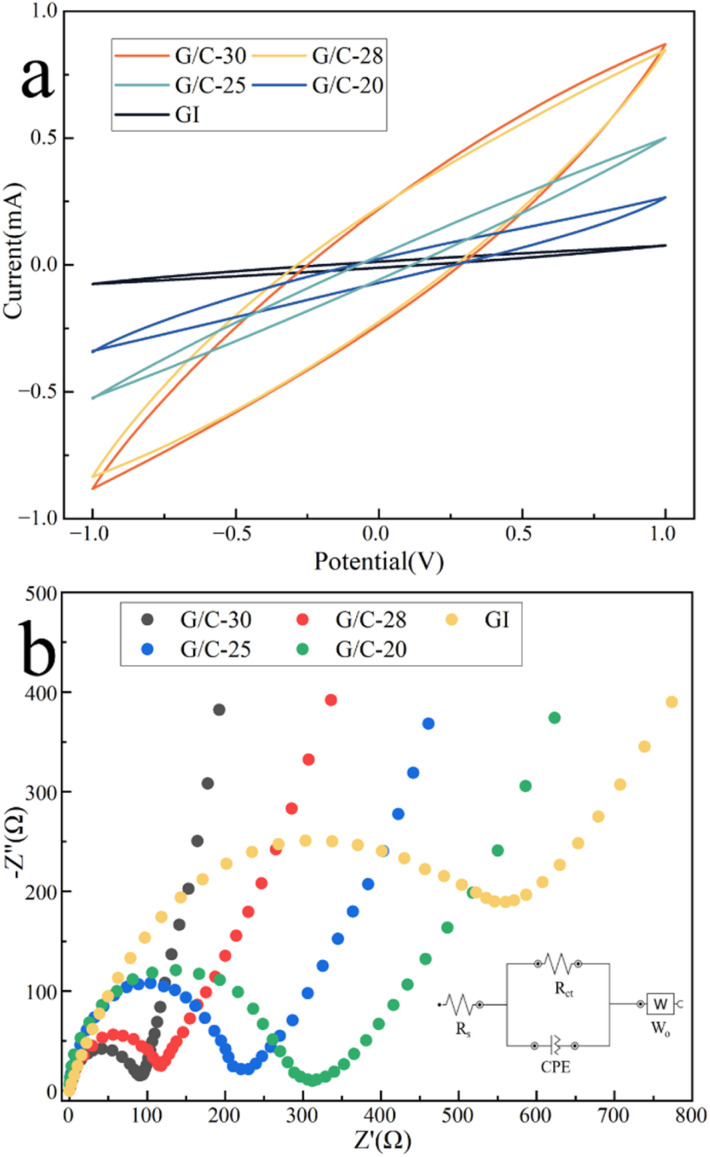
(a) Cyclic voltammetry and (b) electrochemical impedance spectroscopy measurements of G/C-20, G/C-25, G/C-28, G/C-30, and GI ink-printed electrode; (b inset) the equivalent circuit.

Then, the cyclic voltammetry test was done on the screen-printed three-electrode system. We used G/C-20, G/C-25, G/C-28, and G/C-30 ink-printed electrodes, as well as pure graphite-printed (GI) electrodes, in the potential range of −1 V to 1 V in 0.1 M NaCl solution. Fig. 3a illustrates that the higher the conductivity of the electrode, the higher the capacitive current can be obtained. Specifically, at a potential close to 0 V, the capacitive current measured by the G/C-30 electrode is approximately 250 μA, whereas that measured by the GI electrode is only 10 μA, indicating a significant increase in the capacitive current.^41,42^ This is consistent with previous studies that have shown that electrodes modified with nano CB have lower background currents than those modified with carbon nanotubes^42,43^ and graphene oxide,^44^ thus improving sensor measurement accuracy. Furthermore, the composite ink used in this study for printing electrodes has a lower cost compared to materials such as carbon nanotubes and graphene, making it more suitable for large-scale industrial production.

Impedance experiments were conducted on several electrodes across the frequency range of 0.1 Hz to 100 kHz, as illustrated in Fig. 3b. The figure indicates that the electrode produced using G/C-30 ink exhibits a lower charge transfer resistance in the high-frequency region. A lower charge transfer resistance suggests an enhanced charge transfer efficiency of the electrode. Since the electrode created with G/C-30 ink displays a larger capacitive current and a lower charge transfer resistance, it is selected for further investigation.

### Potentiometric characterization of ASS-Na-ISS

**Fig. 4:**
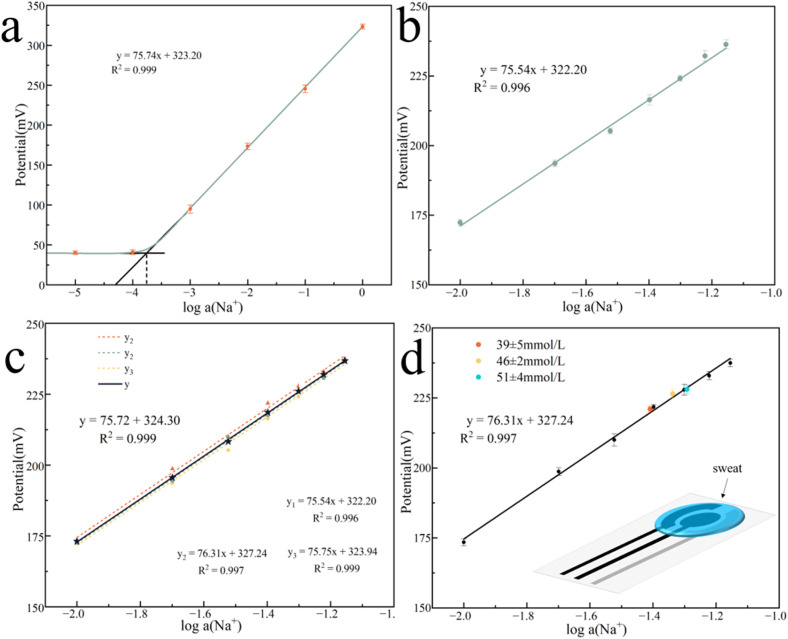
The potential-concentration fitting curve of ASS-Na-ISS in (a) 10^−4^ to 1 M NaCl solution; (b) 10–70 mM NaCl solution; (c) potential–concentration calibration curves of three ASS-Na-ISS in 10–70 mM concentration gradient NaCl solution; (d) ASS-Na-ISS test sweat samples.

We started studying the performance of the ASS-Na-ISS once it was built. In a concentration gradient NaCl solution of 10^−4^ to 1 M, we investigated the ASS-Na-ISS's detecting potential. According to the Nernst equation, the electromotive force (EMF) has a linear relationship with the logarithm of the ion concentration to be measured. Fig. 4a depicts the fitting curve of the ASS-Na-ISS's potential concentration in NaCl solution, with a linear fitting slope of 75.74 mV per decade. The ASS-Na-ISS's limit of detection (LOD) concentration was calculated using the auxiliary line and found to be approximately 0.16 mM, which is much lower than the normal Na^+^ concentration in human sweat (about 40 mM). The ASS-Na-ISS was further calibrated in the range of Na^+^ concentration from 10 mM to 70 mM, as shown in Fig. 4b, based on the typical Na^+^ concentration of human sweat, excluding spots outside the detection limit. In Fig. S4,† the EMF–time response curves obtained by the ASS-Na-ISS in seven concentration gradient NaCl solutions were fitted and calibrated to obtain the curve equation:EMF = 75.54*x* + 322.20, *R*^2^ = 0.996

The repeatability was then assessed using three samples from the same batch (Fig. 4c). After calibration, the slopes of the three sensors were quite comparable, indicating strong repeatability, hence the average value of the three sensors was used to generate the final fitting curve:EMF = 75.72*x* + 324.30, *R*^2^ = 0.999

The steep slope observed in the report is rare, indicating a high sensitivity of the ASS-Na-ISS. To evaluate the usability of the ASS-Na-ISS for practical applications, three sweat samples were collected from participants during exercise, with Na^+^ concentrations of 51 ± 4 mM, 39 ± 5 mM, and 46 ± 2 mM. Fig. 4d was obtained by depositing one of the samples onto the ASS-Na-ISS for the EMF (Fig. S5† shows the EMF–time response curves). The EMF of all three samples were consistent with the fitting results, indicating that ASS-Na-ISS has significant potential in sweat detection.

### Performance evaluation

Aside from sensitivity and detection limit, the ASS-Na-ISS's stability, selectivity, reversibility, and water layer test are all key techniques to evaluate its performance.

#### Selectivity test

**Fig. 5:**
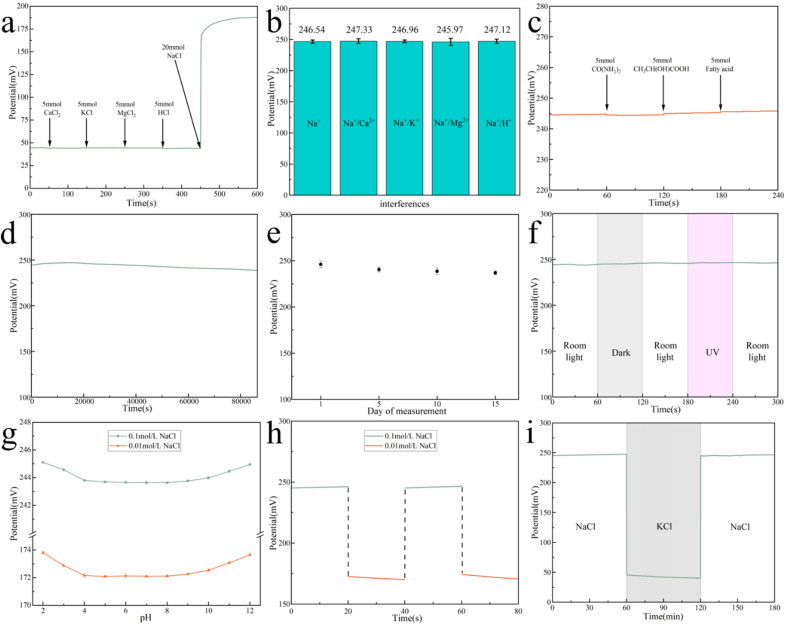
(a) Ion selectivity test; (b) EMF of ASS-Na-ISS under the influence of several ions; (c) EMF of ASS-Na-ISS under the influence of several organic components; (d) long-term test stability; (e) storage stability; (f) photosensitivity; (g) pH sensitivity; (h) reversibility test; (i) water layer test.

The selectivity test for ASS-Na-ISS is performed in an electrochemical reaction cell with rotor stirring, and the potential change is measured by successively introducing the ions under examination. Initially, the test response cell only contains 100 mL of deionized water. The ions are added in the following order: 5 mmol CaCl_2_, 5 mmol KCl, 5 mmol MgCl_2_ and 5 mmol HCl. As shown in Fig. 5a, no noticeable change was observed in the potential curve, indicating that the electrode was not selective for these ions. Immediately after adding 20 mmol of NaCl, the potential curve began to change significantly. Fig. 5b shows the ASS-Na-ISS's EMF in 0.1 M NaCl solution, 0.1 M NaCl and 0.1 M KCl mixed solution, 0.1 M NaCl and 0.1 M CaCl_2_ mixed solution, 0.1 M NaCl and 0.1 M MgCl_2_ mixed solution, and 0.1 M NaCl and 0.1 M HCl mixed solution. As indicated in Fig. 5c, the interference of organic substrates in sweat such as urea, lactic acid, and fatty acids on the sensor has also been examined. It can be seen from these figures that the impact of the presence of these ions and organic substrates on the potential change is within the error range. These results demonstrate that the ASS-Na-ISS is purely selective to Na^+^ and other exogenous cations and organic substrates do not affect electrode sensitivity.

#### Stability

To assess the stability of the ASS-Na-ISS over time, the potential is measured continuously for 24 h in a 0.1 M NaCl solution to verify its operational stability. As shown in Fig. 5d, a fairly consistent signal was detected. The drift potential of the ASS-Na-ISS over 24 h is approximately 0.28 mV h^−1^. Table S2† summarizes several reports from the literature.^1,45–50^ When compared to previous work, the drift potential of the ASS-Na-ISS developed in this study falls within the range reported in these publications. In addition, the ASS-Na-ISS's storage stability was studied. Its storage stability was evaluated by measuring the induced potential on the day of production as well as after 5, 10, and 15 days in 0.1 M NaCl solution, as shown in Fig. 5e. The potential change of the ASS-Na-ISS is still minimal after 15 days of storage at room temperature, and the drift potential is around 0.69 mV per day, showing that the constructed ASS-Na-ISS has high storage stability when maintained dry at room temperature.

#### Photosensitivity

The ASS-Na-ISS's photosensitivity was further examined by exposing it to indoor light, darkness, and UV light and measuring the potential response in 0.1 M NaCl solution. During alternating light source illumination, there was no discernible potential shift (Fig. 5f).

#### pH sensitivity

The pH effect of the ASS-Na-ISS was tested over the range of 2–12 for 0.1 M NaCl solutions and 0.01 M NaCl solutions. The pH was adjusted with HNO_3_ solutions and KOH. The pH sensitivity of the sensor is shown in Fig. 5g. It is clear that the potential was constant in the pH range of 4–9 for both NaCl solutions, which contain the pH range of normal human sweat (4.2–7.5). These data point out that the sensor worked independently of pH changes in the pH range of 4–9.

#### Reversibility

The reversibility of the ASS-Na-ISS was also investigated by varying NaCl concentrations between 0.1 M and 0.01 M. As depicted in Fig. 5h, the effect of concentration changes on potential is minimal, demonstrating the possibility to use the same ASS-Na-ISS for continuous measurements, at several Na^+^ concentrations.

#### Water layer test

The ASS-Na-ISS was also subjected to a water layer test. The presence of a water film between the ISM and the electrode contributes significantly to the measurement instability of the ASS-ISS.^1,51,52^ To determine whether a water film exists, immerse the electrode in a 0.1 M NaCl solution. After 30 min of steady potential, remove the ASS-Na-ISS and immerse it in a 0.1 M KCl solution. Return the potential to the ASS-Na-ISS when it has stabilized for 30 min. In the original NaCl solution, record the time-dependent change curve of the induced potential, as illustrated in Fig. 5i. The detecting potential of the ASS-Na-ISS only displays a minor potential change of 1.17 mV after switching the solution twice, confirming that the water film between the ISM and the electrode has essentially little effect. This might be owing to the strong hydrophobicity of the carbon material used in the printed electrodes, which removes the influence of the water layer and improves ASS-Na-ISS's stability.

## Conclusions

The remarkable electrochemical properties, stability, and cost-effectiveness of carbon nanomaterials have had a significant impact on the advancement of potentiometric sensors. In this study, we have developed a screen-printed sensor for detecting sweat. By harnessing the unique properties of graphite and CB, we have developed highly conductive and eco-friendly inks. The use of this ink in printing the WE lead to sensors that do not form water film, and compared to some of the studies in Table S2,† our sensors exhibit excellent Nernst response, selectivity and stability. Our tests demonstrate that the composite ink based on graphite and CB is well-suited for the preparation of ASS-Na-ISS, and its low cost makes it highly attractive for large-scale production. Furthermore, we have successfully tested the sensor on human sweat samples, which indicates its potential for use in wearable health monitoring devices.

## Author contributions

All authors contributed to the study's conception and design. Material preparation was performed by Wanggang Zhang and Jian Wang. Data collection and analysis and the first draft of the manuscript were performed by Dengke Wang. Review and editing were performed by Xiaohong Li. Yiming Liu provided financial support for this project. All authors read and approved the final manuscript.

## Conflicts of interest

There are no conflicts to declare.

## Supplementary Material

RA-013-D3RA01410J-s001
