# Supplementary material for: A high-performance, all-solid-state Na+ selective sensor printed with eco-friendly conductive ink
Source: RSC Adv. 2023 Jun 6;13(24):16610–8. doi: 10.1039/d3ra01410j (PMC10242244; doi:10.1039/d3ra01410j)
Supplement: RA-013-D3RA01410J-s001 [file RA-013-D3RA01410J-s001.pdf]

Electronic Supplementary Material (ESI) for RSC Advances.
This journal is © The Royal Society of Chemistry 2023

Table S1. ingredient table of the ink

Composition	Mass
Graphite	60g
CB	30g
BYK-022	3g
T-859	6g
Waterborne polyurethane resin	87g
Deionized water	114g

Table S2. Na^+-ISSs in some literature

Sensor	Analyte	Nanomaterial	LOD (M)	Slope (mV/dec)	Drift (mV/h)
V-AuNW electrodes[42]	Na^+	V-AuNW	10^{-3}	58.2	2.2
Paper-based electrode[43]	Na^+	Graphene	10^{-6}	55.7	0.18
PVC-based surfactant sensors[44]	Na-SDS	Plasticisers	10^{-6}	46.4~66.3	0.4~1.2
Flexible ISE[45]	Na^+, K^+	Hydrogel electrolyte	10^{-3}	＜50	——
SPE[1]	Na^+	CB	10^{-4}	58	0.6
OECT with PSSNa electrolyte[46]	Na^+, K^+	OECTs	10^{-5}	84	——
This work	Na^+	Graphite/CB	10^{-4}	75.72	0.28

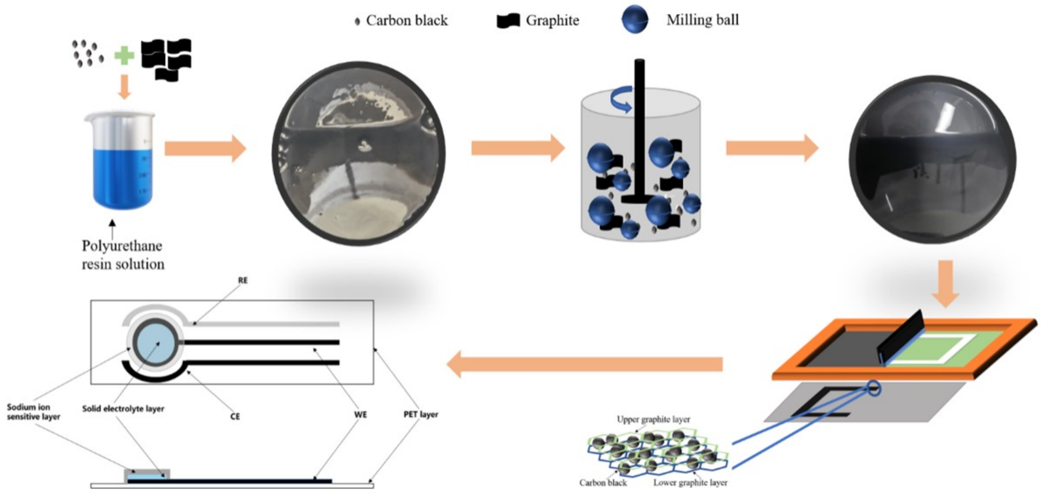

Fig S1. Work flow chart

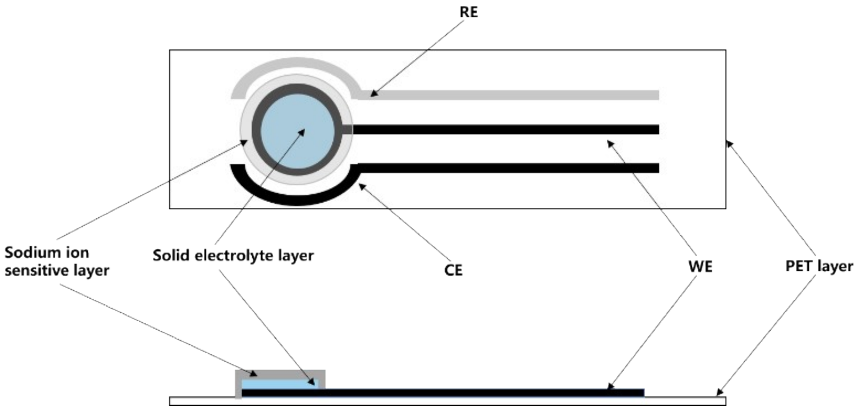

Fig S2. Structure of ASS-Na^+-ISS

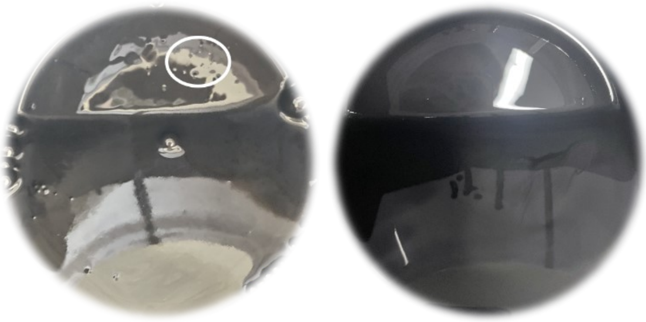

Fig S3. Ball milling causes the disappearance of large particles in the ink. (left) Before ball milling; (right) After ball milling.

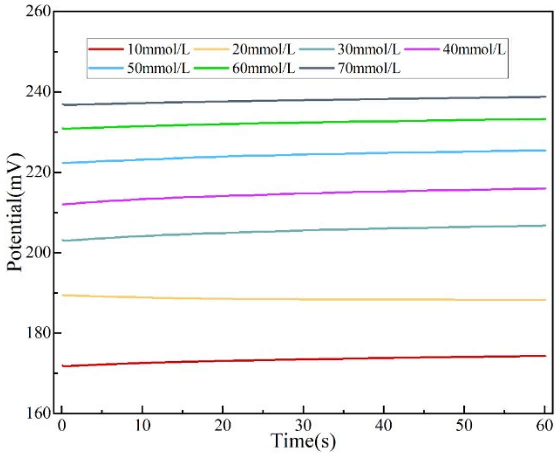

Fig S4. EMF-Time response curves of ASS-Na^+-ISS

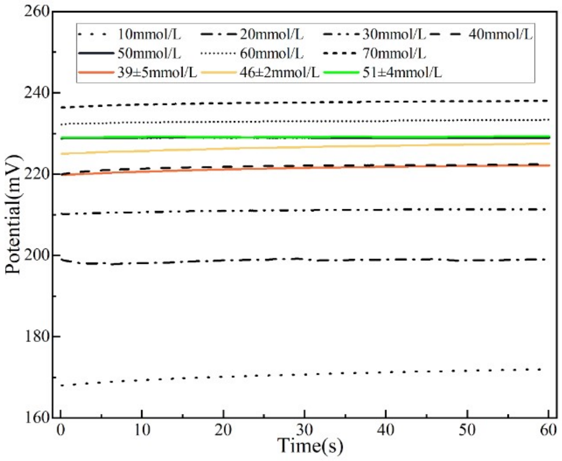

Fig S5. EMF-Time response curves obtained from testing human sweat samples
